# Trained Immunity for Personalized Cancer Immunotherapy: Current Knowledge and Future Opportunities

**DOI:** 10.3389/fmicb.2019.02924

**Published:** 2020-01-10

**Authors:** Joana R. Lérias, Eric de Sousa, Georgia Paraschoudi, João Martins, Carolina Condeço, Nuno Figueiredo, Carlos Carvalho, Ernest Dodoo, Andreia Maia, Mireia Castillo-Martin, Antonio Beltrán, Dário Ligeiro, Martin Rao, Alimuddin Zumla, Markus Maeurer

**Affiliations:** ^1^ImmunoSurgery Unit, Champalimaud Centre for the Unknown, Lisbon, Portugal; ^2^Digestive Unit, Champalimaud Clinical Centre, Lisbon, Portugal; ^3^Molecular and Experimental Pathology Laboratory, Champalimaud Centre for the Unknown, Lisbon, Portugal; ^4^Department of Pathology, Champalimaud Clinical Centre, Lisbon, Portugal; ^5^Lisbon Centre for Blood and Transplantation, Instituto Português do Sangue e Transplantação, Lisbon, Portugal; ^6^Division of Infection and Immunity, NIHR Biomedical Research Centre, UCL Hospitals, NHS Foundation Trust, University College London, London, United Kingdom

**Keywords:** trained immunity, macrophages, dendritic cells, inflammation, cancer, pathogens, immune responses, immunotherapy

## Abstract

Memory formation, guided by microbial ligands, has been reported for innate immune cells. Epigenetic imprinting plays an important role herein, involving histone modification after pathogen-/danger-associated molecular patterns (PAMPs/DAMPs) recognition by pattern recognition receptors (PRRs). Such “trained immunity” affects not only the nominal target pathogen, yet also non-related targets that may be encountered later in life. The concept of trained innate immunity warrants further exploration in cancer and how these insights can be implemented in immunotherapeutic approaches. In this review, we discuss our current understanding of innate immune memory and we reference new findings in this field, highlighting the observations of trained immunity in monocytic and natural killer cells. We also provide a brief overview of trained immunity in non-immune cells, such as stromal cells and fibroblasts. Finally, we present possible strategies based on trained innate immunity that may help to devise host-directed immunotherapies focusing on cancer, with possible extension to infectious diseases.

## Background

Monocytic cells including macrophages and dendritic cells (DCs), granulocytes and natural killer (NK) cells, which feature a spectrum of innate immune cells, constitute the quintessential first line of host innate immune defense and appear to undergo epigenetic reprograming during an antimicrobial immune response (Nakayama et al., 2011; Abbas et al., 2014; Saeed et al., 2014; Vono et al., 2017). The permanent polarization of certain subsets of these cells – triggered by pathogen-driven inflammation – leads to the development of molecular signatures forming an “immunological matrix.” This “trained immunity” does not resemble immunological memory of adaptive immune cells, i.e., T and B cells, but rather pre-programing of cells that will respond with similar effector molecules to subsequent challenge driven by recognition of pathogen-/danger-associated molecular patterns (PAMPs/DAMPs) by pathogen-recognition receptors (PRRs), such as Toll-like receptors (TLRs) (Gourbal et al., 2018). Stroma-associated mesenchymal stromal cells (MSC) and fibroblasts, which can also harbor pathogens (Das et al., 2013; McCormack et al., 2013; Beamer et al., 2014; Khan et al., 2017), are equipped with the capacity to present antigens to T cells via the human leukocyte antigen (HLA) class I and class II pathways during inflammation (Ilangumaran et al., 2002; Romieu-Mourez et al., 2007; Morandi et al., 2008; Das et al., 2013; Crowley et al., 2018; Hamada et al., 2019), and have been discussed to possess trained immunity characteristics (Hamada et al., 2019).

There are several examples of the clinical use of attenuated microorganisms in immunotherapy, such as the attenuated *Mycobacterium bovis* Bacille Calmette-Guerín (BCG) strain as an adjuvant for treatment of non-muscle-invasive bladder cancer (Pettenati and Ingersoll, 2018). BCG induces upregulation of cytokine production, e.g., granulocyte-macrophage colony-stimulating factor (GM-CSF), interleukin-15, tumor-necrosis factor (TNF), expression of MHC class II on urothelial cells and activation of APCs associated with clinically relevant host responses (Ikeda et al., 2002; Mitropoulos, 2005; Bisiaux et al., 2009; Pettenati and Ingersoll, 2018). Clinical studies in Guinea-Bissau have shown that the tuberculosis (TB) vaccine BCG induces cross-protective immune responses among infants in low-resource settings concomitant with a high level of exposure to different infectious agents (Jensen et al., 2015). This is clinically significant, since exposure to a variety of infectious agents early in life in countries with high pathogen transmission rates has been postulated to protect against immunological diseases later in adulthood (MacGillivray and Kollmann, 2014), with a crucial role for PAMP-driven shaping of innate immune responses. Further to the unmistakable role of adaptive immunological memory in immunity, the role of trained immunity in innate immune cells demands attention. In line with this, BCG-primed hematopoietic stem cells (HSCs) – which gave rise to epigenetically modified macrophages – were shown to induce superior recall responses against virulent *Mycobacterium tuberculosis* (*Mtb*) challenge in a mouse model (Kaufmann et al., 2018). Inflammation and HSC plasticity as well as development is similar to immuno-physiological processes occurring in the bone marrow during disease, i.e., TNF-α and IFN-α upregulation and HSC differentiation, G-CSF and IL-1β expression leading to HSC proliferation (Pietras, 2017).

Trained innate immunity may in part be responsible for local fine-tuning and immunomodulation within the bone marrow (and other tissue compartments), where long-term memory T-cell populations can be found in healthy adults (Okhrimenko et al., 2014). Alternatively, initial stimulation of myeloid cells by fungal cell wall-derived β-glucan has been shown to promote superior control of subsequent infection with bacterial pathogens (Quintin et al., 2012; Arts et al., 2018a; Rusek et al., 2018). The role of trained immunity in the context of immunomodulation in cancer was also recently reviewed (Netea et al., 2017), expanding the biological relevance of trained immunity. In this review/viewpoint, we summarize known information concerning trained immunity and discuss relevant observations in view of personalized cancer immunotherapy, particularly on adaptive T-cell responses directed against cancer cells.

## Innate Immune Cells and Immunological Memory

### Macrophages and Dendritic Cells

Priming of human monocytes and monocyte-derived macrophages with LPS, an integral component of bacterial endotoxin (TLR4 ligand), or zymosan, a polysaccharide which belongs to the fungal cell wall (TLR2 and Dectin-1 ligand), has been shown to be cross-reactive (LPS- or -zymosan-primed monocytes can react to either stimulus), albeit with a dependence of the dose of the stimulus (Madej et al., 2017). Importantly, IL-1β production by macrophages initially primed by LPS or *Escherichia coli* is markedly reduced following re-exposure, although in monocytes re-exposure to *E. coli*, but not LPS, produced much higher amounts of IL-1β (Madej et al., 2017). This observation strongly hinted at the exposure of monocytic cells to one type of pathogen affording immune reactivity to another, i.e., bacteria vs. fungi. The immune tolerance induced by LPS could, in part, explain T-cell dysfunction in sepsis syndrome – which is reversible by exogenous IL-7 administration in patients (Francois et al., 2018). A study in mice showed that trained immunity in DC driven by protective vaccination against *Cryptococcus neoformans*, an opportunistic fungal pathogen of the lungs (Kanjanapradit et al., 2017), allowed the trained DCs to generate stronger pro-inflammatory responses against bacterial pathogens *in vitro*, suggesting an effect of trained immunity (Hole et al., 2019).

There is also evidence of *Plasmodium falciparum* (*Pf*)-induced trained immunity in adherent cells from peripheral blood mononuclear cells (PBMCs) – most likely macrophages – which undergo H3K4 trimethylation leading to their subsequent ability to produce high amounts of IL-6 an TNF-α in response to TLR1/2 stimulation with Pam3CSK4 in a manner dependent on hemozoin or *Pf*-infected erythrocytes (Schrum et al., 2018). TLR1 and 2 recognize peptidoglycan, a quintessential component of the bacterial cell wall, and can engage NF-κB activation for pro-inflammatory cytokine signaling, as shown in the context of antimycobacterial immune responses (Takeuchi et al., 2002). As such, TLR1/2-sensisitized macrophages may have a role in the interaction with bacterial pathogens and possibly promote their clearance.

Studies have shown that oxidized low-density lipoprotein particle (oxLDL)- or β-glucan-stimulated macrophages shift to the glycolytic pathway, which promotes polarization to an inflammatory M1 phenotype and induces expression of pro-inflammatory cytokines, such as IL-1β and TNF-α, among others (Biswas, 2015; Groh et al., 2017). Both cytokines have pro- as well as anti-tumor properties in cancer immunology, i.e., priming of T-cell responses and tumor elimination vs. induction of chronic, cancerogenic inflammation (Maeurer et al., 1996; Balkwill, 2009; Gross et al., 2017; Bent et al., 2018; Mantovani et al., 2018). Simultaneously, accumulation of lipids in trained macrophages has been linked to the pathogenesis of atherosclerosis (Groh et al., 2017), raising the question of how much fatty acid metabolism is allowable before it contributes to a different pathology. The shift to aerobic glycolysis in cancer cells fuels their uncontrolled growth, while lactate appears to favor disease dissemination (Jiang, 2017), both of which have also been noted to be necessary for BCG-induced trained immunity in human monocytes (Arts et al., 2016). The implication of this for immunomodulation in cancer requires further assessment.

Subclinical doses of LPS have also been shown to prime and modulate monocyte responses in an interferon regulatory factor 5 (IRF5)-dependent manner, where TIR-domain-containing adapter-inducing interferon-β (TRIF) and TRIF-related adaptor molecule (TRAM), but not Myd88 are involved, following TLR4 activation (Yuan et al., 2016; Geng et al., 2017). Indeed, IRF5-mediated M1 macrophage responses following LPS exposure appear to be necessary for clearing bacterial infections, concomitant with production of reactive oxygen and nitrogen intermediates (Hedl et al., 2018), both of which are necessary in controlling infections but can also promote oncogenesis. The TLR4/TRIF/TRAM pathway is also a currently investigated biological target in cancer immunotherapy (Awasthi, 2014; Guney Eskiler et al., 2019). Agonists of TLR7 have also been found to induce immune tolerance in monocytes at higher doses, while more intense TLR3 stimulation promoted an exacerbated inflammatory response (Geng et al., 2017). Both TLRs recognize RNA structures, suggesting pathogen-derived nucleic acids as a potent inducer of trained immunity, with RNA-based cancer vaccine adjuvants having been shown to induce tumor rejection and anti-viral responses without or with only minimal off-target toxicity (Seya et al., 2015; Zhu et al., 2017; Ziegler et al., 2017). In addition to TLRs, other PRRs such as melanoma-differentiation antigen 5 (MDA-5) and retinoic acid-inducible gene I (RIG-I), largely involved in antiviral defense, have also been implicated in mediating tumor-cell apoptosis, DC priming and potentiation of anti-cancer cytotoxic T-cell activation (Wu et al., 2017).

A recent review by van der Heijden et al. (2018) appraised the role and significance of epigenetic modifications in innate immune cells to establish trained immunity (van der Heijden et al., 2018). Infection with *Mtb*, an intracellular pathogen which prefers to reside in alveolar macrophages, has been shown to induce epigenetic changes in the host cell, i.e., modification of histones 3 and 4 acetylation patterns to promote its prolonged survival (Esterhuyse et al., 2015; Moores et al., 2017; Singh et al., 2017). Furthermore, *Mtb* also triggers the synthesis of host microRNA species to modulate immune responses to its benefit (Iannaccone et al., 2014; Huang et al., 2015; Kumar et al., 2015; von Both et al., 2018). Whether *Mtb*-infected macrophages (and DCs) can modulate immune responses associated with cancer or other infections remains yet to be explored. One study has shown that infection of macrophages with *Mtb* H37Rv, a virulent, laboratory-adapted strain, upregulated PD-L1 expression which lead to increased Treg infiltration into lymph nodes and exacerbated disease in NSCLC-bearing mice (Zhou et al., 2017). It is important to be able to visualize how *Mtb* exposure of monocytic cells in humans may predispose them to either control or succumb to exacerbated inflammation, which may promote cancer in some individuals, and warrants thorough investigation due to the worrying global burden of TB (World Health Organization [WHO], 2018).

Another interesting point is the impact of microbial products in affecting tumor-associated macrophages (TAM), which have been reported as pro-tumoral, promoting angiogenesis, tumor-invasion, metastasis, and fine-tuning tumor-associated inflammation (Esposito et al., 2004; Qian and Pollard, 2010; Szebeni et al., 2017). The TAMs can be originated from circulating monocytes that will enter the tissue and differentiate into macrophages, bone-marrow-derived macrophages (BMDMs) or can result from an accumulation of tissue-resident macrophages (TRMs) (Pathria et al., 2019). Indeed, there is a crescent number of reports correlating TAMs with higher tumor grade and shorter survival for breast cancer, renal cell carcinoma, glioblastoma, pancreatic cancer, head and neck cancer, and lymphoma (Zhang et al., 2013, 2018; Pedersen et al., 2014; Tiainen et al., 2015; Wang et al., 2015; Hu et al., 2016; Atanasov et al., 2018; Gartrell et al., 2018; Sorensen et al., 2018; Pathria et al., 2019). The relationship between TAMs and the tumor invasiveness and ability to metastasis is suggested to be related to epithelial-mesenchymal transition (EMT) (Su et al., 2014; Fu et al., 2015; Ravi et al., 2016). Indeed, Fu et al. (2015) showed that EMT hotspots in hepatocellular carcinoma were associated with TAMs infiltration (Fu et al., 2015). However, TAMs and invasiveness are certainly affected by other factors, e.g., N-cadherin and Snail (Helm et al., 2014; Lin et al., 2019).

Nevertheless, the reacquisition of proinflammatory characteristics in macrophages, so called repolarization, was associated with increased survival in mice and patients with different cancer types and may be a future approach for cancer therapy (Kaneda et al., 2016b; Pathria et al., 2019). Two recent studies reported that the inhibition of phosphatidylinositol-3-kinase (PI3K) by genetic depletion or pharmacological inhibition, lead to proinflammatory expression in TAMs, with a downstream effect in T-cell activation (Kaneda et al., 2016a, b). The authors also identified that a downstream effect would be to promote NF-kB phosphorylation and DNA binding activity, therefore increasing proinflammatory gene expression associated to such pathway. Another effect is the activation of Bruton’s tyrosine kinase (BTK), which inhibition by ibrutinib stimulates macrophage polarization, myeloid cell infiltration reduction and increase in CD8 + T cells infiltration in murine pancreatic ductal adenocarcinoma (PDAC) (Gunderson et al., 2016). Another molecule associated to the composition of tumor microenvironment effects is the growth arrest specific 6 (Gas6), since it interacts with TAM receptors Mer (Lew et al., 2014), with the downstream effect of PI3K, ERK, and NK-kB pathway activation. Interestingly, overexpression of Gas6 was described in a wide variety of cancers, such as melanoma, schwannoma, glioblastoma, and PDAC (Ito et al., 2002; Hutterer et al., 2008; Song et al., 2011; Demarest et al., 2013; Ammoun et al., 2014). There are other molecules that may be targeted to address the TAMs repolarization, such as receptor-interacting serine/threonine kinase 1 (RIPK1) or Janus kinase 2/signal transducer and activator of transcription 3 (Jak2/Stat3). The first one is increased in TAMs in human PDAC and its inhibition will repolarize TAMs and increase MHC class II, TNF-α and INF-γ expression besides reducing tumor growth (Wang et al., 2018). Besides, RIPK1 inhibition will also activate CD8 + T cells, increase differentiation of CD4 + T cells toward a Th1 phenotype and may have a synergic action with anti-PD-1 antibody (Wang et al., 2018). Regarding Jak2/Stat3, inhibition of Stat3 also leads to repolarization of TAMs and increases infiltration of cytotoxic T lymphocytes (CTLs), which could also be achieved by targeting Jak2 (upstream activator) (Pathria et al., 2015). TAMs may also express a molecule named macrophage receptor with collagenous structure (MARCO), involved in the recognition of PAMPs and TLRs linking innate immune responses in the tumor – microenvironment to pathogens (Mukhopadhyay et al., 2011; Kissick et al., 2014). Indeed, TLR agonists polarize macrophages toward a proinflammatory phenotype, therefore also having a possible role for cancer therapy. The downside of such agonists is the concomitant expression of PD-L1 in macrophages, which could be blocked by the synergistic use of anti-PD-1 antibodies (Kaneda et al., 2016a, b).

### NK Cells

Recent translational studies using human material have shed more light on the molecular changes in “memory-like” NK cells and ways to identify them. Hypomethylation of AT-rich interaction domain 5B (ARID5B) and co-expression of CD57, NKG2C, and reduced CD56 mark an “adaptive” subset of NK cells (Cichocki et al., 2018). Viral infection of NK cells induces the expression of natural cytotoxicity receptors (NCRs), such as NKp46, NKp44 [a HLA-DP401 ligand, which is also associated with tumor recognition (Odunsi et al., 2007; Straetemans et al., 2012; Laheurte et al., 2016; Lu et al., 2017; Niehrs et al., 2019)] and NKp30 as well as the NKG2D receptor, which binds to the non-classical HLA class I-associated molecules MICA/B on tumor cells (Cantoni et al., 2015). Particularly, human CMV infection may also drive the expansion of adaptive NK-cell populations phenotypically characterized as FcεRγ^–^, tyrosine kinase SYK^–^, EAT-2^–^ and master transcription factor PLZF^low^, with reduced IL-12 and IL-18 responsiveness connected to PLZF downregulation (Schlums et al., 2015). CMV-experienced FcγRIII/CD16^+^ NKG2C^+^ memory-like NK cells also undergo Syk DNA hypermethylation, but retain responsiveness to antibody-mediated cell expansion via CD16 binding upon exposure to CMV-infected target cells (Lee et al., 2015). As in mice, memory-like, intrahepatic NKG2C^+^ CD49a^+^ DX5^–^ NK cells co-expressing CD25 and IgG-like receptor, have also been described in humans (Peng et al., 2013; Marquardt et al., 2015). A recent translational study showed that “trained,” intrauterine NK-cell populations with epigenetic modifications in the IFN-γ and VEGF-A and high propensity to produce these cytokines following stimulation loci might play an important role in successful placentation (Gamliel et al., 2018). Functional studies in mice revealed that virus-induced memory-like NK cells, after contraction, go on to reside in lymphoid and non-lymphoid organs and are able to facilitate enhanced viral control following adoptive transfer (Sun et al., 2009). Thus, tissue-derived NK cells may have specific trained immunity features which are of biological relevance not only in cross-protective immunity but also tissue physiology.

The generation of memory-like NK cells has also been demonstrated by exposing them to a combination of IL-12, IL-15 and IL-18, referred to as cytokine-induced memory-like (CIML) NK cells, resulting in a population of effector cells which also exhibits superior control of K562 leukemia cells (Leong et al., 2013; Rosario et al., 2014). It is important to note that these cytokines are also produced by macrophages and DCs as a first-line immune armament during infection (Abbas et al., 2014). As such, additionally to pathogen-derived stimuli, the local cytokine milieu may also promote immunological memory in NK cells in tissue. Taking these observations into consideration, how pathogen-driven formation of immunological memory in NK cells would affect tumor immunosurveillance warrants formal testing using appropriate models and may be very significant for clinical immunotherapy.

## Non-Immune Cells and Trained Immunity

Trained immunity in non-immune cells has been appreciated and extensively reviewed elsewhere (Hamada et al., 2019). Regulation of trained immunity in MSCs by microRNA expression and DNA methylation has been demonstrated following LPS exposure, where pro-inflammatory cytokine expression was maintained even in the absence of stimulus (Liu et al., 2015). Fibroblasts are highly specialized cells required for immune signaling during infection and tissue repair following inflammation-induced cell damage, making them a potential drug target to ameliorate chronic inflammation (Flavell et al., 2008). Their expression of TLRs and close interaction with surrounding and infiltrating immune cells places fibroblasts at an important axis linking trained immunity and immunopathology (Miteva et al., 2014). For instance, sustained activation of the Twist1-Prrx1-TNC PFL in cancer-associated fibroblasts (CAFs) perpetrates fibrotic lesions during idiopathic pulmonary fibrosis (Lederer and Martinez, 2018; Yeo et al., 2018). Tissue fibrosis, impairment of organ function and immune-suppression are also reminiscent of pulmonary TB (Dheda et al., 2005) and solid tumors (Jiang et al., 2016), hinting at similar mechanism at play. Intestinal stromal cells have been previously described to provide long-lasting pro-inflammatory immune responses against pathogens further to recruiting immune cells to the site of infection (Owens, 2015). Transformed cells, stromal cells and fibroblasts also provide a rich source of growth factors, pro-tumorigenic and immune-suppressive cytokine production that facilitates tumor progression (Todoric and Karin, 2019). Suitable disease models and well-defined clinical samples are necessary to address the role of trained non-immune cells in the cross-reactive immune responses in infectious diseases and malignant transformation.

## Can Trained Immunity Be Exploited for Therapeutic Purposes?

**FIGURE 1:**
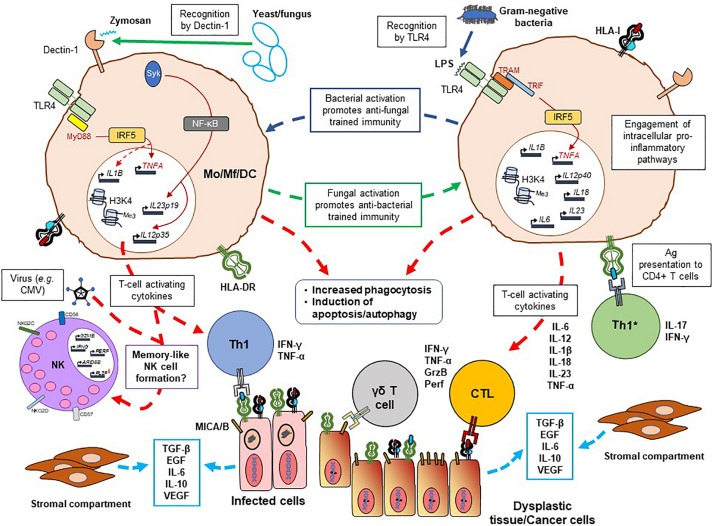
Possible immunological processes potentiated by trained innate immunity against infections and cancer. The schematic represents some of the cardinal immune mechanisms at play in establishing trained immunity in monocytic cells, including macrophages and DCs. Bacteria-/fungi-exposed monocytes or macrophages (in response to bacterial LPS or fungal zymosan detected by pathogen recognition receptors TLR4 and Dectin-1, for example) can cross-react to either pathogen type with the production of pro-inflammatory cytokines (IL-6, TNF-α, IL-1β, IL-18, IL-12, and IL-23) which are necessary for T-cell activation. Myd88 and/or TRIF/TRAM-mediated IRF5 activity appears to play an important role herein, along with epigenetic modification (largely involving methylation patterns) of histone 3 at position lysine 4 (H3K4). Antigen presentation via the HLA class I and class II (HLA-DR is shown in the schematic) and the inflammatory tissue environment would promote priming and expansion of T helper, cytotoxic T lymphocyte (CTL) and TCR γδ T-cell populations. These T cells can eliminate infected cells and control the spread of the pathogen, and possibly react to dysplastic cells which may develop due to inflammation-driven genetic aberrations. Similarly, there may also be a direct effect of the innate immune cells – trained by exposure to pathogens – to produce an inflammation-driven response to future infections as well as cancer cells. The latter may express both classes of HLA molecules, only the TCR γδ ligands MICA/B (which also binds to NKG2D on NK cells as well as γδ T cells), a combination of the two or HLA class II alone in the event of mutational events leading to HLA class I loss. Ongoing inflammation in tissue leads to the production of several cytokines and growth factors (e.g., TGF-β, EGF, IL-6, IL-10, and VEGF) by infected cells, dysplastic tissue and cells in the stromal compartment (activated stem cells, fibroblasts), potentially exerting a pro-tumor effect and impairing the host’s anti-tumor response. The cytokine microenvironment and the possible infection with virus, such as CMV, may also promote the development of memory NK cells in tissue. Mo, monocyte; Mf, macrophage; DC, dendritic cell; HLA, human leukocyte antigen; LPS, lipopolysaccharide; IRF5, interferon regulatory factor 5; IFN-γ, interferon gamma; TNF-α, tumor necrosis factor alpha; GrzB, granzyme B; perf, perforin; TLR, Toll-like receptor; TRIF, TIR-domain-containing adapter-inducing interferon-β; TRAM, TRIF-related adaptor molecule; Me, methyl group; TGF-β, transforming growth factor beta; EGF, epidermal growth factor; VEGF, vascular endothelial growth factor; NK, natural killer cell.

While trained immunity may induce unwanted, pathological inflammation and, therefore, constitutes an avenue of pharmacological intervention (Mourits et al., 2018; Mulder et al., 2019), its utility in shaping the repertoire of antigen-specific/antigen-experienced immune cells may be useful against different diseases indicates an element of “in-built adjuvanticity.” In a recently reported phase 1 clinical study, BCG-vaccinated individuals given a dose of *P. falciparum* were shown to afford better control of malaria, concomitant with early activation of granzyme B^+^ NK cells and HLA-DR^+^ monocytes (Walk et al., 2019). Non-vaccinated controls did not show similar results, suggesting that BCG-driven innate immune activation leads to cross-protection against a protozoan parasite, in keeping with a previous finding describing pro-inflammatory, adherent innate immune cells responses due to plasmodium-triggered, trained immune responses (Schrum et al., 2018). In another study, Arts et al. (2018b) reported that BCG vaccination would induce a genome-wide epigenetic reprograming of monocytes. Epigenetic changes due to BCG vaccination involved G protein-coupled receptors and protein kinases, and several signaling pathways involved in cytokines and chemokines production, such as the PI3K/AKT (phosphatidylinositol 3-kinase) pathway, epidermal growth factor receptor (EGFR), fibroblast growth factor (FGF), and vascular endothelial growth factor (VEGF) signaling pathways. The translation of this epigenetic reprograming was a higher pro-inflammatory cytokine production (TNF-α, IL-1β, and IL-6) of PBMCs from vaccinated, as compared to placebo-treated individuals, emphasizing the impact of trained immunity. Besides, these immune changes would also confer a higher protection to an unrelated infection (yellow fever virus), due to a higher production of IL-1β, and as a trained immunity response (Arts et al., 2018b). Another interesting aspect was described by Buffen et al. (2014), reporting that BCG induced trained immunity in monocytes with an unrelated stimulus, measured by increase of IL-6 and TNF-α cytokines, would not occur when autophagy was blocked. Indeed, both pharmacological inhibition of autophagy or single nucleotide polymorphisms (SNPs) in the autophagy genes (ATG2B and ATG5) reduced the trained immunity effect of BCG, due to the blocking of epigenetic reprograming of monocytes at H3K4 trimethylation (Buffen et al., 2014). Besides, the authors also describe an increase rate of recurrence and progression of non-muscular invasive bladder cancer patients after intravesical instillations of BCG in patients who exhibited SNP in the autophagy gene ATG2B. This observation supports the importance of the genetic background in non-specific effects of BCG in trained immunity and argues for genetic analyses of tissue material from patients undergoing BCG installation. Other pathogens affect as well immune cells. *C. neoformans* was shown to produce prostaglandin E2 (PGE2) to suppress T-cell activation for promoting its own growth and survival in macrophages (Evans et al., 2019). PGE2 has several important anti-inflammatory effects encompassing the TNF/IL-6/IL-17 axis and IL-8 production by inducing epigenetic modifications (Venza et al., 2012; Adamik et al., 2013; Harizi, 2015). Whether microbe-trained PGE2 production by memory-like monocytic cells may have a biologically relevant role in ameliorating chronic inflammation has to be elucidated. IFN-γ- and LPS (TLR4)-primed macrophages, although capable of superior phagocytosis of apoptotic lymphoma cells compared to non-primed macrophages, were skewed toward an M2 (anti-inflammatory phenotype) and exhibited pro-tumor effects *in vivo* (Voss et al., 2017) in a preclinical (murine) model. It is, however, unknown whether TLR activation driven by factors in the tumor microenvironment (such as bacteria or fungal commensals, please see below) can promote trained immunity and, if so, whether such innate immune memory help control transformed cells and/or pathogens (see Figure 1).

## Personalized Cancer Immunotherapy

Natural killer cell-mediated immune reactivity – particularly in hematological malignancies and in combination with chimeric antigen receptor (CAR) expression – forms a central structure in cancer immunotherapy (Bjorklund et al., 2018; Tang et al., 2018). A highly favorable characteristic of NK cells pertinent to clinical use is that they are obtainable from allogeneic sources for therapy, can mediate graft-versus-leukemia (GvL) responses (Locatelli et al., 2018) and are amenable to *in vitro* conditioning to acquire memory-like properties (Iliopoulou et al., 2010; Liang et al., 2017). NKG2A, the NK cell/CD8^+^ T cell-expressed interaction partner for HLA-E on targets cells, represents a new immune checkpoint molecule which has already shown therapeutic potential in several preclinical cancer models (Tognarelli et al., 2018; van Montfoort et al., 2018; Creelan and Antonia, 2019). NK-cell exposure to CMV induces NKG2A upregulation albeit not compromising the cells’ ability to produce IFN-γ (Petersen et al., 2010). NKG2A^+^ memory-like NK cells may, therefore, be clinically beneficial for cellular therapy of patients with HLA-E^hi^ malignancies (de Kruijf et al., 2010; Benevolo et al., 2011; Gooden et al., 2011; Lin et al., 2011; Bjorklund et al., 2018). CMV may also imprint on anti-cancer directed immune responses, which may be of clinical relevance, since CMV as well as EBV-reactive T- and B-cells infiltrate into tumor lesions (Meng et al., 2018; Lerias et al., 2019). Reprograming of tumor-associate T-cells by epigenetic targeting of CD8 + tissue resident memory (Trm) cells and tumor infiltrating T-lymphocytes (TIL) may also promote tumor control, in part by increasing “mitochondrial fitness” (Li et al., 2019).

Modulation of histone methylation using pharmacological agents has been proposed as a potential host-directed strategy to capitalize on trained innate immunity to provide immune protection (Mulder et al., 2019; Rodriguez et al., 2019). Among the crucial host proteins involved in histone methylation is lysine demethylase 6B (KDM6B), also known as Jumonji Domain-containing 3 (JMJD3). LPS activation of macrophages, a cardinal early event in sepsis, leads to downstream mobilization of several KDM6B targets, especially those associated with pro-inflammatory responses (De Santa et al., 2009). A similar effect is true for serum amyloid protein A (SAA)-driven inflammatory responses in macrophages (Yan et al., 2014), which is linked to the pathogenesis of rheumatoid arthritis and potentially cancer as well as metastasis (Liu, 2012; Zhou et al., 2018; Lee et al., 2019). KDM6B expression is linked to better prognosis in patients with neuroblastoma (Yang et al., 2019) and stabilization of the tumor suppressor protein p53 in glioblastoma stem cells (Ene et al., 2012) while its loss has been shown to promote pancreatic cancer-cell aggressiveness (Yamamoto et al., 2014). Thus, the role LPS-triggered “training” of macrophages via its effect on KDM6B warrants further elucidation in the context of personalized cancer medicine.

Conversely, with respect to helminth infections, KDM6B along with IRF4 triggers the anti-inflammatory reprograming of macrophages (M2 phenotype), downstream of which manifests in Th2 cytokine release and antibody production (Satoh et al., 2010). These observations hint at the pleiotropic nature of KDM6B engagement in modulating host macrophage function as an essential therapeutic target to protect against a myriad of extrinsic (pathogen-associated) and intrinsic (host-associated) insults. Interestingly, amino acids 1110-1120 of KDM6B contain a strong 8 amino-acid match with selected residues between positions 251–265 in the influenza A virus (H1N1) hemagglutinin (HA) protein (derived from the California/New York strains of the 2009 pandemic flu), which provides a small hint about molecular mimicry and the possibility of TCR binding. Further studies are necessary to understand how pre-programing of KDM6B activity affects disease outcome in infectious diseases.

The microbiome has an important role in promoting trained immunity due to effect in development of the immune system, host control of chronic infections (e.g., TB), and clinical responses to immune checkpoint blockade in cancer for developing next-generation personalized cancer immunotherapies (Nash et al., 2017; Cassone, 2018; Gupta et al., 2018; Fessler et al., 2019). Indeed, gut microbial/non-microbial ligands are essential for the adaptative immunity during secondary infection/pathogenic exposures, being involved in the production of immunomodulatory metabolites, such as short-chain fatty acids or secondary bile acids, regulating innate immune cells metabolism and functions (Kitahara et al., 2001; Tremaroli and Backhed, 2012; Levy et al., 2016; Rooks and Garrett, 2016; Jia et al., 2018). Importantly, commensals in the gut are involved in the production of immunomodulatory metabolites that comprise short-chain fatty acids (SCFAs) such as butyrate, acetate, and propionate (50–52). Further, commensals such as *Bacteroides*, *Lactobacillus*, and *Bifidobacteria species* synthesize secondary bile acids that are derived from the metabolism of primary bile acids (53–55). Binding of these bioactive molecules to the receptors on the innate cells regulate their metabolism and functions (Negi et al., 2019). Cancer associated microbiomes have recently been linked to clinical outcomes in pancreatic cancer: The mycobiome (fungal components of the microbiome) has been shown to accelerate pancreatic cancer, via a carbohydrate moiety on *Malassezia* that activates the complement pathway (Aykut et al., 2019). In contrast, the tumor microbiome characterized by *Saccharolpolyspora*, *Pseudoxanthomonas*, *Bacillius clausii* and Streptomyces species has been associated with long-term survival for patients with pancreatic cancer (Riquelme et al., 2019). Future studies will show the impact of these bacterial/fungal species and their metabolites on immune cell programing.

Cancer antigens are released into the external environment usually by dying cells or packaged in exosomes (Wolfers et al., 2001). This may (i) facilitate training of immune cells and help them respond to a future infection or other cancer indications or (ii) activate immune cells subsets which are pre-wired – by a previous infection or exposure to autoantigens – to exhibit enhanced phagocytic functions, cytokine production capacity and unleash strong anti-tumor T-cell responses (Netea et al., 2017). Indeed, the durable changes after training of innate myeloid cells, involve the increase of expression and release of cytokines associated to a long-term regulation of gene transcription through epigenetic mechanisms (Foster et al., 2007; Quintin et al., 2012; Netea et al., 2017). More specific effects of trained immune cells is, for example, the switch from oxidative phosphorylation to glycolysis in trained monocytes (Cheng et al., 2014). Besides, trained monocytes also show the accumulation of fumarate in the Krebs cycle, inhibiting the KDM5 family of H3K4 demethylases, therefore ensuring the maintenance of the H3K4me3 open chromatin mark (Sun et al., 2015).

Dendritic cells -based vaccination constitutes a major area of targeted personalized immunotherapy, with naturally occurring circulating DCs with certain pre-programed characteristics being considered of value for therapeutic applications (Bol et al., 2019). Herein, trained immunity in DCs – such as that shown in response to anti-*C. neoformans* vaccination (Hole et al., 2019) – warrants investigation in the context of tailored anti-cancer immune responses. The DC vaccines involve the ability of these cells to act as an antitumoral effector in both CTLs and NK cells, in order to eradicate malignant cells (Kirkwood et al., 2012). There are several types of DC-vaccines, being the most frequently used the reinfusion of *ex vivo* derived DC pulsed with tumor-associated antigens (TAAs) or tumor cell lysates and stimulated with TNF-α, IL-1β, IL-6, and prostaglandin E2 (PGE2) (Lee et al., 2002; Koski et al., 2008; Anguille et al., 2014). The DC-based immunotherapy efficiency may be enhanced using immune checkpoint inhibitors, such as anti PD-1 or anti-CTLA-4 antibodies (Mastelic-Gavillet et al., 2019). Carreno and colleges described the vaccination of three stage III resected melanoma patients who received mature autologous DCs pulsed with peptides derived from mutated antigens, with a previous treatment with CTLA-4 blockade. Interestingly, besides the identification of peptide-specific T cell responses, after vaccination blood samples showed a more diverse TCR repertoire (Carreno et al., 2015). DC vaccines can also be considered to be combined with chemotherapy, since it is reported that chemotherapy may deplete specific cell types, such as Tregs and myeloid derived suppressor cells (MDSCs) and modulate the immune system to a more pro-inflammatory state (Kershaw et al., 2013; Bracci et al., 2014).

## Conclusion

With increasing evidence emerging from basic and translational studies, trained immunity warrants further dissection for its capacity to offer powerful and durable anti-cancer immune responses – and potential reprograming of “non-productive” to “productive” (i.e., anti-cancer or pathogen-directed) immune responses. A large repertoire of innate immune and non-immunes cells enriches the repertoire of responders to insults of various origins and nature, and their interplay in shaping immunity. Combining biomarker information from various clinical studies and drug trials will increase the possibilities for designing treatment strategies. Trained immunity-based approaches will inevitably enhance T-cell responses in conferring host protection and facilitating long-term adaptive memory responses against pathogens or transformed cells.

## Author Contributions

MR, JL, AZ, and MM wrote the first draft and conceptualized the review. All authors were involved in further development, writing and proofreading of the review.

## Conflict of Interest

The authors declare that the research was conducted in the absence of any commercial or financial relationships that could be construed as a potential conflict of interest.
